# Determination of fungal diversity of acidic gruel by using culture‐dependent and independent methods

**DOI:** 10.1002/fsn3.1844

**Published:** 2020-10-19

**Authors:** Yurong Wang, Mina She, Zhuang Guo, Quan Shuang

**Affiliations:** ^1^ College of Food Science Inner Mongolia agricultural university Hohhot China; ^2^ Northwest Hubei Research Institute of Traditional Fermented Food College of Food Science and Engineering Hubei University of Arts and Sciences Xiangyang China

**Keywords:** acidic gruel, fungal diversity, high‐throughput sequencing, yeast

## Abstract

Traditional fermented cereals are a rich source of naturally derived, diverse microorganisms. Illumina MiSeq high‐throughput sequencing was used to investigate thoroughly fungal microflora in Western Inner Mongolian acidic gruel. A total of 589,495 sequences were obtained from 16 acidic gruel samples. Ascomycota was found to be the predominant phylum with a relatively abundance of 97.54%, followed by Basidiomycota (2.26%) and Chytridiomycota (0.1%). The dominant genera obtained from the acidic gruel were *Candida*, *Galactomyces*, *Hanseniaspora*, *Guehomyces*, *Zygosaccharomyces*, *Trichosporon*, *Rhodosporidium*, *Penicillium,* and *Blastobotrys*. *Candida* and *Galactomyces* were predominant genera, and their relative abundances were 57.59% and 34.95%, respectively. A total of 50 yeast strains were isolated and identified. Statistical analysis indicated that *P kudriavzevii* and *Geo. silvicola* affiliated with Ascomycota were the dominant yeasts in acidic gruel, accounting for 28% and 22%, respectively. This study provides an unequivocal theoretical basis for the study of fungal diversity and the identification and preservation of yeasts in traditional fermented cereals. It also provides validated strain resources for further exploration of the effect of yeasts on acidic gruel quality.

## Graphical abstract

Diverse fungal species were abundant in traditional fermented acidic gruel, and significant differences between different samples were seen; The core fungal genera in acidic gruel were Candida and Pichia; Pichia kudriavzevii and Geotrichum silvicola were the dominant yeasts in acidic gruel.

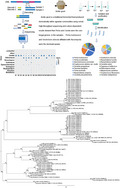

## 1 INTRODUCTION

Fermentation has been a successful method of preservation, with an estimated consumption in excess of 5,000 varieties of fermented food globally (Johansen, 2018; Tamang, Koichi, & Holzapfel, 2016). Raw materials for fermentation include cereals, vegetables, fruits, milk, and meat; among which, cereal fermented products are very popular throughout the world (Gänzle & Salovaara, 2019). Acidic gruel is a traditional fermented food produced domestically within agrarian communities using rice, millet, glutinous rice, and other types of cereal. Raw material is first boiled and then cooled, before the residual sour broth is placed in a jar at room temperature overnight. The fermented acidic gruel can be used to make fried sour rice or sour porridge (Wang, Zhang, Zhang, et al., 2010). Millet was the main material used in acidic gruel from Inner Mongolia, Shanxi, and Shaanxi. Its protein content is similar to wheat, but the essential amino acid content is higher, and it also contains a variety of vitamins and rich mineral elements, indicating the high nutritional value of millet consumption. At the same time, it is one of the traditional Chinese herbal medicines in China, which has certain medicinal value (Wei, Lina, Wenhao, Baoxin, & Longkui, 2019; Zhang et al., 2011). Since the time of Song Dynasty, Inner Mongolia, Shaanxi, Shanxi, and Guangxi have habitually made and eaten acidic gruel, foods made with it can calm both the stomach and spleen and reduce fever and elevated body temperature, but is yet to achieve industrial production. Research on acidic gruel has proved fruitful. Li, Chang, and Wang (2016) and Chang (2016) used pure culture approach to isolate and identify the yeast and lactic acid bacteria in acidic gruel from Northwest Shanxi, while Qin (Qin et al., 2019) and Zhang (Zhang, Li, Song, & Luo, 2020) explored traditional acidic gruel preparation on the basis of strains, fermentation temperature, time, and amount of physalis. However, most studies have focused on the separation and identification of lactic acid bacteria and yeast by traditional culture methods or explored acidic gruel production under different conditions. There are few reports detailing the comprehensive analysis of fungal diversity within traditional fermented acidic gruel.

Fungi are essential microorganisms in the production of Chinese fermented, with different fungi are present in different raw materials and production methods, contributing for example to the unique flavors and texture of traditional acidic gruel (Stephanie, Burton, & Reid, 2015). Many studies have highlighted the importance role which yeasts play in the fermentation products. They also have an important impact on food preservation include inhibiting of the growth of spoilage microorganisms (Angmo, Kumari, Savitri, & Bhalla, 2015; Zhang, 2010). Further study of fungal microorganisms in acidic gruel using advanced technology may offer an enhanced understanding of its intrinsic microbial community and improve both the production process and the ultimate quality of acidic gruel.

Many advanced molecular ecology methods have been developed and used widely to analyze rapidly and efficiently microbial communities in fermented food (Liang, Yin, Zhang, Chang, & Zhang, 2018; Sun et al., 2018). Illumina MiSeq is a powerful and attractive molecular biological technique able to evaluate microbial diversity (Nutan, Changotra, Grover, & Vashistt, 2018; You et al., 2016). It is simple to use, low in cost, and highly suitable for use in small labs (Melanie et al., 2015). High‐throughput sequencing technology has been used widely to study the microbial diversity of fermented foods. However, there are few relevant studies of the fungal diversity of acidic gruel.

In this study, Illumina MiSeq sequencing was used to evaluate the fungal microbial communities in acidic gruel, and traditional microbial culture was used to isolate and identify yeasts. The objective of this research was to analyze the diversity of fungal microflora in acidic gruel. It also provides a suitable basis for further study of the microbial community within it and thereby improves the quality of acidic gruel.

## 2 MATERIALS AND METHODS

### 2.1 Samples collection and DNA extraction

Research team collected 16 samples of traditional fermented acidic gruel from different households in Ordos, Bayan Nur, and Baotou in western Inner Mongolia. These samples were numbered A1‐A16. The raw material in the samples was primarily millet. After washing, six times the volume of water was added, and the resulting traditional fermentation gruel soup was left as primer to ferment for 24 hr at room temperature. The fermented mature acidic gruel was milky white, the physalis was thick, and the pH value was approximately 4.0.

Total genomic DNA was extracted from acidic gruel samples used the QIAGEN DNeasymericon Food Kit (QIAGEN, Germany) following to the manufacturer's instructions. The integrity of genomic DNA was analyzed using 0.8% (w/v) agarose gel electrophoresis in 1 × TAE buffer. DNA concentration was measured using a Micro‐ultraviolet spectrophotometer (Nano Drop 2000c, Thermo, America).

### 2.2 18S rDNA PCR amplification and sequencing

The total volume of the 26S rDNA polymerase chain reaction (PCR) amplification was 20 μl. This comprised 4 μl 5 × PCR Mix, 2.5 μl dNTP mix (2.5 mmol/L), 0.8 μl SSU0817F (5’‐TTAGCATGGAATAATRRAATAGGA‐3’) (5 μmol/L), 0.8 μl SSU1196R (5′‐TCTGGACCTGGTGAGTTTCC‐3′) (5 μmol/L), 0.4 μl Taq DNA polymerase (5 U/μl), and 10 ng DNA, made up with ddH_2_O to a total volume of 20 µl. Seven barcodes were added to the forward primer. PCR amplifications used the following protocol: predenaturation at 95°C for 3 min, then 30 cycles of denaturation at 95°C for 30 s, annealing at 55°C for 30 s, extension at 72°C for 45 s, and a final extension for 10 min at 72°C. PCR products were analyzed using 0.8% (w/v) agarose gel electrophoresis, diluted to 100 nmol/L, and then sequenced using the MiSeq Sequencing Platform (Illumina, America).

### 2.3 Sequences analysis

Raw data were initially filtered and merged to remove barcodes, primers, adapters, and failed fragments (Liu & Tong, 2017; Tanja & Steven, 2011). Trimmomatic (v0.35) and Usearch (v8.1) were used to obtain high quality sequences (Bolger, Marc, & Bjoern, 2014). Aligned sequences were clustered into operational taxonomic units (OTUs), using 1.00 and 0.97 as similarity thresholds within UCLUST (Edgar, 2010). Representative sequences were selected from each OUT. SILVA (Quast et al., 2012) was used to assign these sequences to their respective phylum, class, family, order, and genus (Cole et al., 2013). The α‐diversity of 26S rDNA sequences from the samples including Chao 1, the observed species, and the Shannon and Simpson indices calculated using the QIIME platform (Caporaso et al., 2010) Cluster analysis was undertaken using weighted UniFrac distances and variance analysis (Hamady, Lozupone, & Knight, 2009).

### 2.4 Isolation and identification of yeasts

Gradient dilution was used to process samples using sterile physiological saline solution (0.15% peptone, 0.85% NaCl). Three gradients (10^–1^, 10^–2^, 10^–3^) were coated on PDA agar (6% potato powder, 20% glucose, 20% agar, pH = 5.6 ± 0.2) (Griffith et al., 2007) for 72–120 hr at 28°C. Characteristic colonies were selected. Single colonies were obtained using the streak plate method.

Genomic DNA was isolated using the modified procedure of Fujimori (Fujimori & Okuda, 1995). PCR amplifications using the following protocol: predenaturation at 94°C for 4 min, then 30 cycles of denaturation at 95°C for 1 min, annealing at 50°C for 45 s, extension at 72°C for 1 min, and a final extension for 10 min at 72°C. The PCR amplification volume was 25 μl. The primers used were NS1 (5′‐GCATATCAATAAGCGGAGGAAAAG‐3′) and NS4 (5′‐GGTCCGTGTTTCAAGACGG‐3′) (Wang & Bai, 2009). The resulting PCR products were purified, cloned, and sequenced by Tianyi Huiyuan Biological Technology Co., Ltd. (Wuhan, China). The phylogenetic tree of yeasts was made using BioEdit (v7.1.3), DNAMAN (v8.0), and IQ‐TREE (v1.6.2).

### 2.5 Accession numbers of Nucleotide sequences

Raw sequence data have been made publically available online through MG‐RAST (http://www.mg‐rast.org/mgmain.html?mgpage=project&project=mgp91622). The project number was mgp91622. Nucleotide sequences of evaluated yeasts have been deposited at GenBank with accession numbers MH880134‐MH880183.

## 3 RESULTS AND DISCUSSION

### 3.1 Sequence data analysis and diversity by high‐throughput sequencing

After filtering and preprocessing, a total of 589,495 sequences were obtained from 16 traditional fermented acidic gruel samples, 10,895 different OTUs were obtained under 97% similarity. The abundance and diversity of acidic gruel are reflected by Chao 1 (representing species richness), Simpson index (representing species diversity), and Shannon index (Table 1). The Chao 1 index of sample A16 (5,850) was higher than that of other samples, and the observed species of sample A3 (1,192) was the highest. Sample A7 (0.81) had the highest Simpson index, while sample A15 (3.89) had the highest Shannon index. These results indicate that the fungal abundance of samples A16 and A3 was the highest among the samples, investigated. The fungal diversity of samples A7 and A15 was the highest. The abundance and diversity of sample A13 were the lowest, seen. This is consistent with it having low fungal diversity.

**TABLE 1:** 18S rRNA sequencing and analysis of fungal microbiota from acidic gruel samples: total sequences, OTUs, taxonomic classifications at different levels, and quality indices

*N*.O.	Sequences	OTUs	Phylum	Class	Order	Family	Genus	Chao 1	Observed species	Shannon	Simpson
A1	31,474	776	3	11	11	29	25	3,177	765	2.64	0.45
A2	30,740	1,161	2	2	3	2	6	3,421	1,160	1.25	0.17
A3	32,427	1,235	3	1	13	18	23	3,018	1,192	1.25	0.17
A4	43,458	1,090	2	4	3	12	18	3,252	843	2.34	0.47
A5	32,521	781	2	11	11	19	26	4,419	748	2.82	0.69
A6	38,193	899	2	7	6	13	15	3,789	763	1.75	0.36
A7	30,936	850	4	11	19	34	53	3,325	846	3.77	0.81
A8	37,695	1,365	4	7	5	7	8	3,185	1,186	1.24	0.17
A9	44,020	1,005	2	10	10	18	27	3,290	750	0.94	0.15
A10	43,588	959	2	6	8	18	18	2,138	716	1.24	0.23
A11	34,605	1,280	2	10	9	24	19	3,959	1,181	1.58	0.25
A12	34,362	1,240	2	7	6	8	8	3,234	1,150	1.33	0.19
A13	43,048	913	2	3	1	9	7	3,399	678	0.76	0.13
A14	42,157	1,388	2	6	4	11	6	3,082	1,125	1.17	0.16
A15	31,998	1,098	3	9	7	13	19	3,752	1,068	3.89	0.78
A16	38,273	1,287	3	10	10	14	20	5,850	1,105	2.43	0.59
Mean ± *SD*	36,843 ± 5,072	1,083 ± 205	3 ± 1	7 ± 3	8 ± 5	16 ± 8	19 ± 12	3,518 ± 792	955 ± 204	1.90 ± 0.97	0.36 ± 0.24

Note

When calculating the Chao 1, Observed species, Shannon and Simpson index of each sample, the total sequences from the samples were 30,710.

**FIGURE 1:**
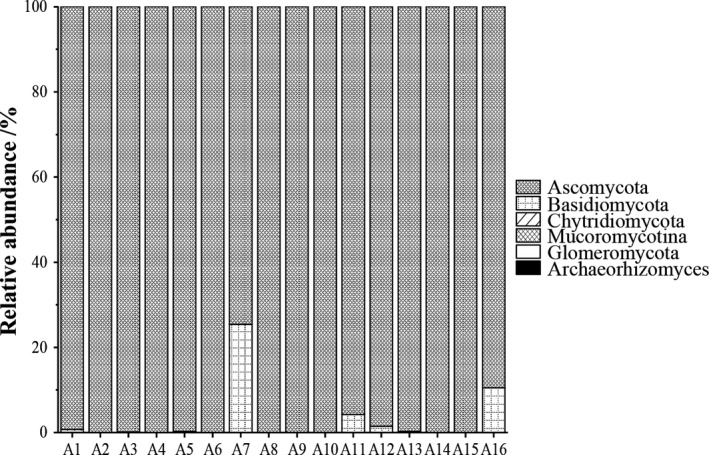
Comparative analysis of the relative abundance of fungal phyla in acidic gruel. A fungal phylum with average relative content >1% was defined as a dominant phylum. Fungal phyla with an average relative content of <1% were defined as others

**FIGURE 2:**
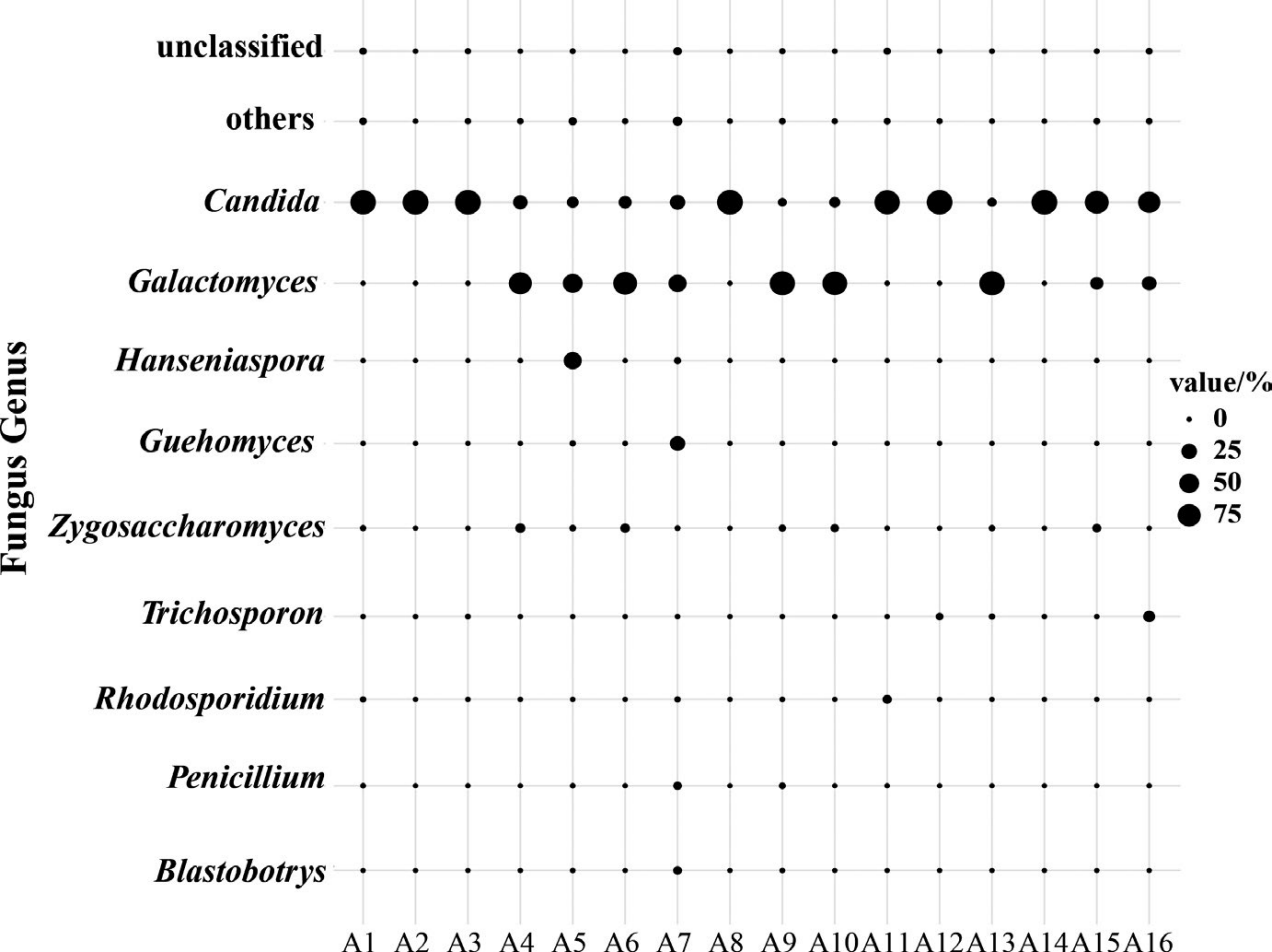
Comparative analysis of the relative abundance of dominant fungal genera in acidic gruel. A fungal genus with an average relative content >1% was defined as dominant genus. Fungal genera with an average relative content of <1% were defined as others

All of sequences were classified into 6 phyla, 15 classes, 30 orders, 52 families, and 85 genera. Ascomycota, Basidiomycota, and Chytridiomycota were identified as dominant fungal phyla (Figure 1). Among these three phyla, Ascomycota (97.54%) was the most abundant group, followed by Basidiomycota (2.26%) and Chytridiomycota (0.1%). These results indicate Ascomycota to be the predominant fungal phylum in acidic gruel. A total of 9 main genera were detected, with only 0.37% being unclassified. Those with relative abundance less than 0.1% were merged with other genera (Figure 2). The average relative abundance of each genera in acidic gruel was as follows: *Candida* (57.59%), *Galactomyces* (34.95%), *Hanseniaspora* (2.43%), *Guehomyces* (2.43%), *Zygosaccharomyces* (1.06%), *Trichosporon* (0.75%), *Rhodosporidium* (0.26%), *Penicillium* (0.22%), and *Blastobotrys* (0.19%). *Candida* was the dominant fungal genus in samples A1, A2, A3, A8, A11, A12, A14, A15, and A16, with relative abundance of 97.12, 99.87, 99.40, 99.93, 94.91, 98.51, 99.85, and 99.95%, respectively.*Galactomyces* was the dominant fungal genus in the sample of A4, A5, A6, A7, A9, A10, and A13, with relative abundance of 74.31, 49.37, 81.29, 38.75, 94.58, 89.99, and 95.40%, respectively. The average relative abundance of *Pichia* was 0.09%, which was detected in all samples, as was *candida*. At the OTU level, a total of 10,895 OTUs were obtained in acidic gruel; OTU6112, OTU3445, OTU3125, OTU1398, OTU1273, and OTU10189 were the most dominant OTUs (relative abundance more than 1.0%) in the acidic gruel; and their average relative abundance was 38.56, 33.70, 8.54, 1.75, 1.36, and 1.35%, respectively. However, the relative abundance of each dominant OTUs in different samples was different. OTU6112, OTU3125, and OTU10189 were similar to *Candida*, OTU1398 was similar to *Hanseniaspora,* while OTU1237 was similar to *Tausonia*. These relationships were consistent with the results of the analysis shown in Figure 2.

Taking three acidic gruel samples from the Ordos region of Inner Mongolia as examples, the previous study found the dominant fungal phyla to be Ascomycota (96.46%) and Basidiomycota (3.53%) (Wang & Xing, 2017; Y. Wang, Zhang, Lei, et al., 2010). It also indicated that *Candida* (84.30%)*, Galactomyces* (11.86%), and *Trichosporon* (3.45%) to be the dominant genera. These findings were similar to those of the present study. *Candida* found in natural fermentation products (Banjara, Suhr, & Hallen‐Adams, 2015; Wang & Xing, 2017) contributes to the production of special flavours and to the growth of acid producing lactic acid bacteria (Gadaga, Mutukumira, & Narvhus, 2000). Previous work indicates that *Candida* species produce a large number of lipases during food fermentation (María et al., 2005) which is vital for the proper formation of flavor and aromatic esters(Gupta, Kumari, Syal, & Singh, 2015; Sillers et al., 2017).

### 3.2 Sample difference analysis

**FIGURE 3:**
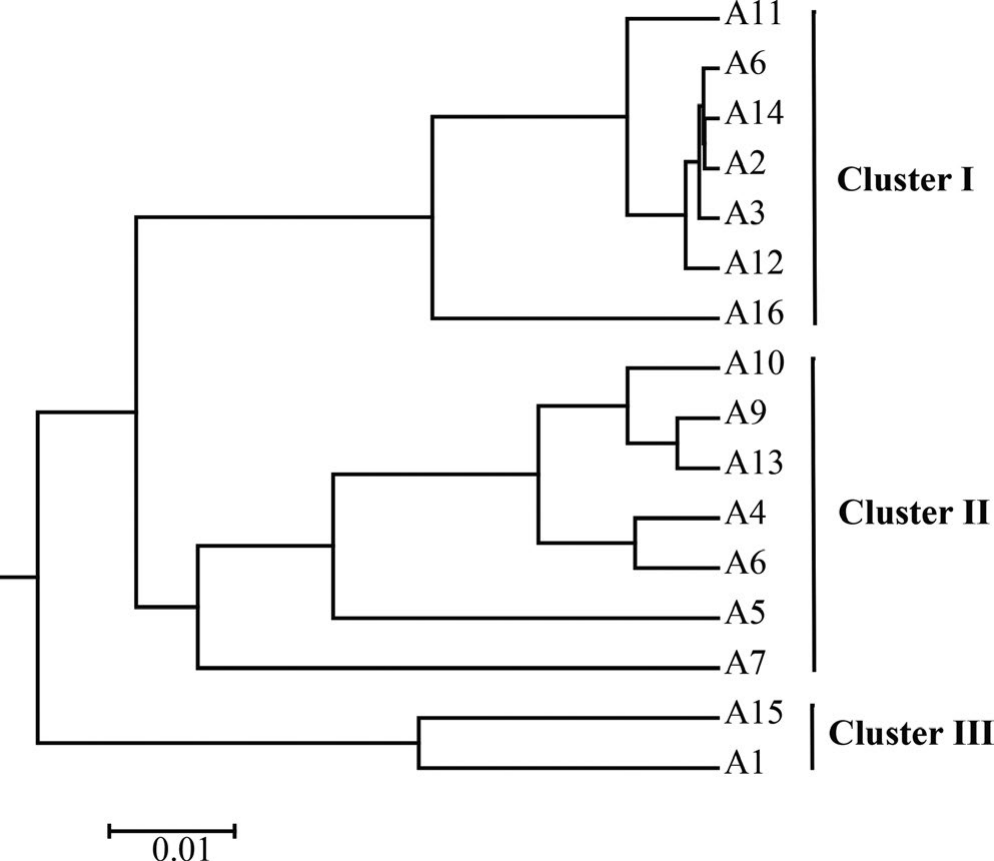
Cluster analysis of OTUs based on weighted UniFrac distance. The distance between branches and between clusters indicates the evolutionary distance between samples

The cluster analysis based on the weighted UniFrac distance metric indicated differences for all samples (Figure 3). Taking sequence abundance into account to further quantify the variation in different lineages between samples, it was found that the 16 samples of acidic gruel could be classified into three clusters. Cluster I contained 7 samples (A2, A3, A8, A11, A14, and A16). Sample A4, A5, A6, A7, A9, A10, and A13 formed cluster II, while cluster III contained only sample A1 and A15. The results indicate that the fungal community of each cluster was different. As the number of samples in cluster III was small, only samples from cluster I and cluster II were analyzed for variance. The main fungal genera causing differences between the two clusters were *Galactomyces* and *Candida* (*p* < .01) (Table 2).

**TABLE 2:** Correlation analysis of OTUs based on weighting UniFrac distance

Genus	Cluster I	Cluster II	*p* value
*Galactomyces*	2.97 (0,0–20.75)	74.81 (81.29,38.75–95.4)	.0015
*Candida*	94.37 (99.4,67.94–99.97)	11.73 (9.62,3.04–23.75)	.0017
*Hanseniaspora*	0 (0,0–0.01)	5.55 (0.02,0–37.89)	.0627
*Guehomyces*	0 (0,0–0.01)	3.43 (0.01,0–23.72)	.0735
*Zygosaccharomyces*	0 (0,0–0.01)	1.96 (0.96,0.1–5.19)	.0111
*Trichosporon*	1.66 (0.01,0–10.27)	0.04 (0,0–0.28)	.2284
*Rhodosporidium*	0.51 (0,0–3.53)	0.05 (0,0–0.3)	.7093
*Penicillium*	0 (0,0–0.01)	0.48 (0.01,0–2.68)	.1548
*Blastobotrys*	0 (0,0–0.01)	0.44 (0,0–3.01)	.1737

Note

Data in the table expressed as an average (median, minimum – maximum). Type III contains two samples. Calculating its mean and median would have no meaning in statistics, so was not shown.

### 3.3 Isolation and identification of yeast

**FIGURE 4:**
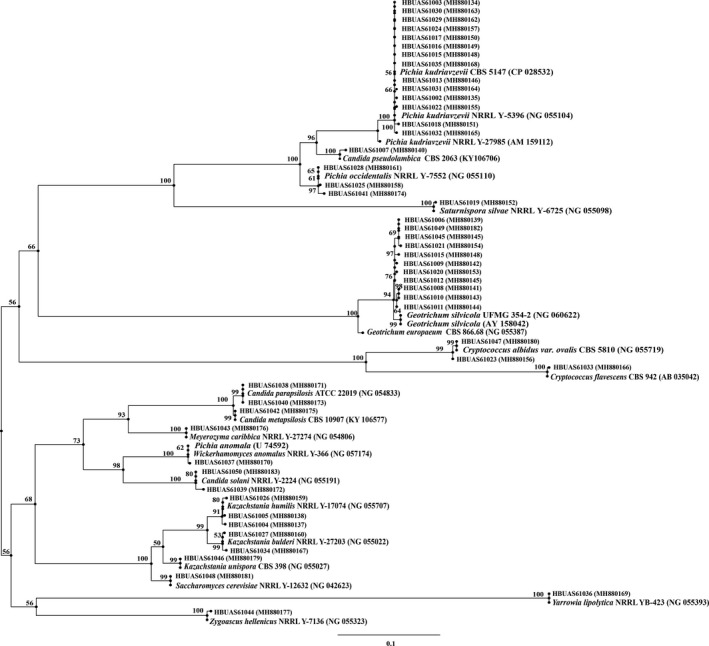
Phylogenetic tree of yeasts identified in acidic gruel. The similarities of all strains to their standard strains were all over 99%. Serial numbers beginning HBUAS refer to yeasts isolated in this study

Based on the above analysis, it was found that the cumulative relative content of the fungal genera belonging to yeasts in the detected fungal genera exceeds 98%, and 6 out of the 9 dominant fungal genera were yeasts, indicating that yeasts in the traditional fermented acidic gruel were rich and varied. The method based on combination of cultivation‐dependent and molecular biology technology was used to identify the yeast from 16 acidic gruel samples, isolating a total of 50 yeasts. A phylogenetic tree was derived, and it depicts the genetic relationship between 50 yeast strains isolated from the 16 acidic gruel samples. This comparison shows that 18 strains were similar to the genus of *Pichia*, eleven strains belonged to *Geotrichum*, seven strains belonged to *Candida*, six strains belonged to *Kazachstania*, two strains belonged to *Cryptococcus,* and two strains belonged *Saccharomyces*. The remaining four strains belonged to *Geotrichum*, *Saturnispora*, *Yarrowia,* and *Zygoascus*, respectively (Figure 4). There were differences observed between yeast isolation results and the fungal diversity analysis. This may be due to the limitations of traditional microbiological methods and the specific growth of particular strains. Conventional pure culture methods can only detect approximately 10% of microorganisms from environmental samples, while Illumina MiSeq requires only 0.05–1.0 μg of DNA template, can be amplified, and can detect noncultivable and low‐abundance microorganisms.

**FIGURE 5:**
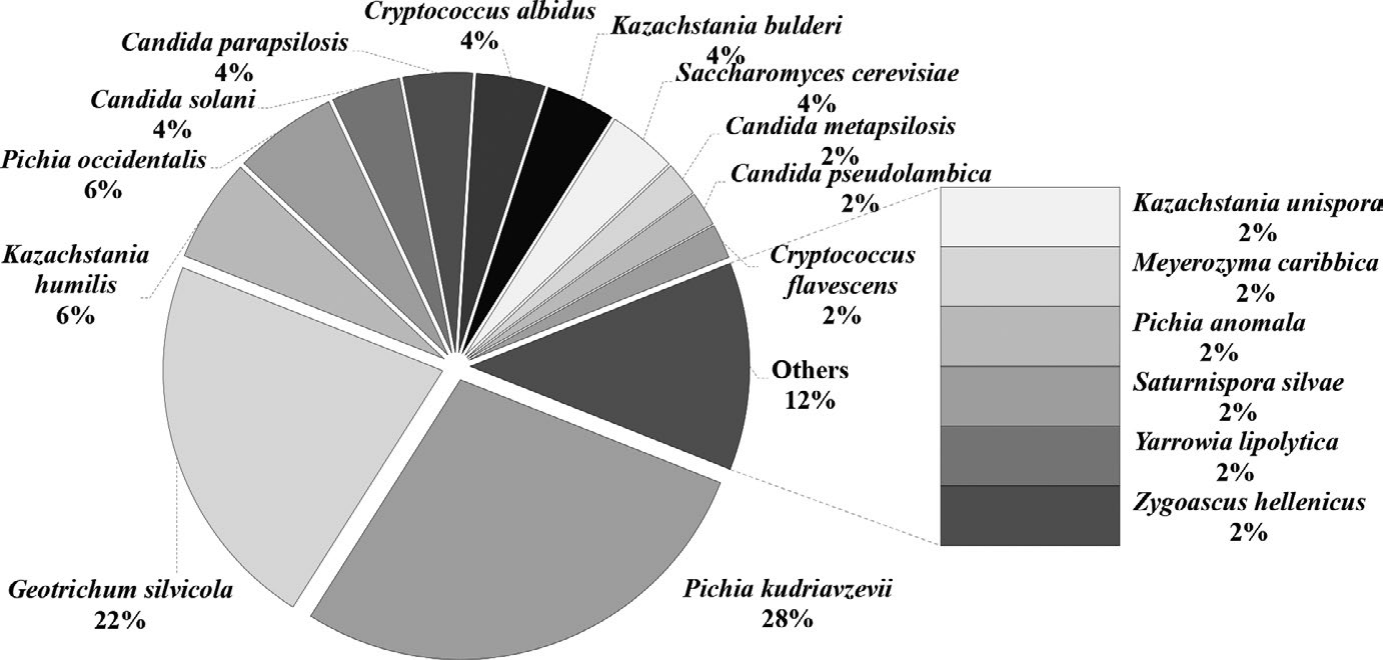
The relative abundance of all yeasts isolated from acidic gruel. 50 yeast strains were isolated from the samples of acidic gruel analyzed in the study

According to the statistical analysis, *Pichia kudriavzevii and Geotrichum silvicola* were the dominant yeasts, with relative abundance of 28% and 22%, respectively (Figure 5). Followed by *Pichia occidentalis* and *Kazachstania humilis*, each accounting for 6%.*Candida parapsilosis, Candida solani, Cryptococcus albidus, Kazachstania bulderi,* and *Saccharomyces cerevisiae* all each accounted for 4%. The proportion of *Candida metapsilosis, Candida pseudolambica, Cryptococcus flavescens, Kazachstania unispora, Meyerozyma caribbica, Pichia anomala, Geotrichum silvicola, Yarrowia lipolytica,* and *Zygoascus hellenicus* were all 2%. *Galactomyces* was also one of the dominant fungal genera, but strains belonging to *Galactomyces* were not isolated from acidic gruel. This may be due to the growth conditions or inherent limitations of pure culture approach. *P kudriavzevii* (previously called *C krusei*) plays a crucial role in the production of traditional fermented food, and it may have potential probiotic effects (Greppi et al., 2013, 2017; Pedersen, Owusu‐Kwarteng, Thorsen, & Jespersen, 2012). Other work suggests *P kudriavzevii and Geo. silvicola* can also enhance food flavor and improve the freshness of food. Eric (Grondin et al., 2017) evaluated the ability of 30 strains of yeast belonging to *Dipodascus*, *Galactomyces*, *Geotrichum*, *Magnusiomyces,* and *Saprochaete* to produce volatile flavor substances with HSSPME‐GC/MS analysis and found that *Geotrichum* has good ester production capacity. Citric acid metabolism is an advantage of *P kudriavzevii*, which can reduce the acidity of cocoa pulp, promote the endogenous proteolysis of raw materials and other enzymatic activities in the process of cocoa fermentation, thereby affecting the quality of cocoa beans and chocolate and possibly affecting the microbial ecology of the entire fermentation process (Soccol et al., 2017).

Traditional methods used to produce fermented food are greatly affected by weather, temperature, and microorganisms in the environment, resulting in the inconsistent taste and flavor of food. To understand its diversity and function of microorganisms in the production of acidic gruel, several researchers have studied yeast. Bai (Bai et al., 2010)isolated 40 yeast strains from 28 traditional fermented acidic gruel samples from western Inner Mongolia. The dominant species were *I orientalis* (75%, frequency percentage), *S cerevisiae* (14.29%), *Geotrichum sp*. (10.71%), *C pararugosa* (7.14%), *C parapsilosis* (7.14%), *T asahii* (7.14%), *T coremiiforme* (3.57%), *C tropicalis* (3.57%), and *Cla. lusitaniae* (3.57%). Using DGGE technology, *T asahii*, *S cerevisiae*, *Geotrichum sp*., and *I orientalis* were detected in acidic gruel from western Inner Mongolia (Zhang, 2010). In a recent study, a total of 41 yeast strains were isolated from six fermentation stages of northwestern Shanxi acid gruel (Li et al., 2016). Physiological and biochemical experiments combined with ITS4 and ITS5 analysis showed that all strains could be classified into 5 types according to their morphological characteristics. These had similar it to *P* *kudriavzevii* (99%, homology), *I orientalis* (100%), and *S cerevisiae* (99%). Such studies indicate that elucidation of fungal diversity within acidic gruel and the accurate of yeast has been a longstanding research interest and that clear differences arise from analysis of different regional samples and the use of different research methods. This is a principal reason for using MiSeq high‐throughput sequencing technology, so that the fungal diversity in acidic gruel can be analyzed rigorously and completely. As well as acidic gruel, the researchers also investigated fungi and yeasts found in similar traditional fermented cereal foods from different regions and countries. *Idil* is a traditional fermented food from India and Sri Lanka, *S cereuisiae*, *Debaryomyces hansenii*, *Hansenula anomala,* and *T beigelii* were common yeasts involved in the fermentation of *Idil* (Durgadevi & Shetty, 2014; Soni & Sandhu, 1989). *Ogi,* made from maize, sorghum, or millet and produced by fermenting maize grains, is popular in several West African countries (Adegoke & Babalola, 1988).*S cerevisiae*, *C krusei*, *Geo. candidum, Geo. fermentans, C tropicalis,* and *R graminis* were isolated during *ogi* fermentation (Omemu, Oyewole, & Bankole, 2007). *S cerevisiae*, *C pelliculosa,* and *Candida tropicalis* have also been isolated from t*ogwa*, a Tanzanian fermented food (Mugula, Nnko, Narvhus, & Sørhaug, 2003). These innate mycological communities are similar to those found in this study. However, for the yeast species isolated, there were appreciable differences between *I orientalis* and *S cerevisiae*. These may be caused by differences in local geography, and climate, as well as grain types and producers.

## 4 CONCLUSIONS

To summarize, this work was a first approach to analyze the fungal community structure of traditional fermented Inner Mongolian acidic gruel with high‐throughput sequencing and pure culture technology. Multivariate statistical analysis indicated that the fungi in acidic gruel were abundant. Although there were certain differences between the samples, *Pichia* and *Candia* were the core fungal genera. Some in the isolated yeasts were found to have potential utilization value. Future research will further characterize these and other yeast strains, while also exploring the mechanism underlying how these and other fungi influence the physical and chemical properties of acidic gruel.

## CONFLICT OF INTEREST

The authors have declared no conflict of interest.
